# Sensorimotor Learning of Acupuncture Needle Manipulation Using Visual Feedback

**DOI:** 10.1371/journal.pone.0139340

**Published:** 2015-09-25

**Authors:** Won-Mo Jung, Jinwoong Lim, In-Seon Lee, Hi-Joon Park, Christian Wallraven, Younbyoung Chae

**Affiliations:** 1 Acupuncture and Meridian Science Research Center, College of Korean Medicine, Kyung Hee University, Seoul, Korea; 2 Department of Brain and Cognitive Engineering, Korea University, Seoul, Korea; Tokai University, JAPAN

## Abstract

**Objective:**

Humans can acquire a wide variety of motor skills using sensory feedback pertaining to discrepancies between intended and actual movements. Acupuncture needle manipulation involves sophisticated hand movements and represents a fundamental skill for acupuncturists. We investigated whether untrained students could improve their motor performance during acupuncture needle manipulation using visual feedback (VF).

**Methods:**

Twenty-one untrained medical students were included, randomly divided into concurrent (*n* = 10) and post-trial (*n* = 11) VF groups. Both groups were trained in simple lift/thrusting techniques during session 1, and in complicated lift/thrusting techniques in session 2 (eight training trials per session). We compared the motion patterns and error magnitudes of pre- and post-training tests.

**Results:**

During motion pattern analysis, both the concurrent and post-trial VF groups exhibited greater improvements in motion patterns during the complicated lifting/thrusting session. In the magnitude error analysis, both groups also exhibited reduced error magnitudes during the simple lifting/thrusting session. For the training period, the concurrent VF group exhibited reduced error magnitudes across all training trials, whereas the post-trial VF group was characterized by greater error magnitudes during initial trials, which gradually reduced during later trials.

**Conclusions:**

Our findings suggest that novices can improve the sophisticated hand movements required for acupuncture needle manipulation using sensorimotor learning with VF. Use of two types of VF can be beneficial for untrained students in terms of learning how to manipulate acupuncture needles, using either automatic or cognitive processes.

## Introduction

Humans can control their motion during behaviors ranging from simple limb movements to complex, delicate motor skills. We can acquire a wide variety of motor skills every day, and adapt to changes even in unfamiliar environments [1]. Sensorimotor learning is based on sensory feedback pertaining to discrepancies between desired and actual movements [2]. Humans can estimate the error gradients of each motor command component, and improve performance through iterative corrections based on movement error [3]. Information concerning movement error plays a crucial role in motor learning [4]. Both concurrent and post-trial visual feedback (VF) improved motor performance during an isometric target acquisition task in two different ways: concurrent VF enhanced motor performance through automatic recalibration of visuomotor mapping, while post-trial VF induced improvements using a cognitive strategy [2].

Acupuncture needle manipulation requires sophisticated hand movements and represents a fundamental skill for acupuncturists. Acupuncture experts consolidate motion patterns during needle manipulation by accumulating clinical experience [5]. Experts exhibit greater kinematic and kinetic movement consistency and temporal efficiency during needle manipulation compared with novices [6,7]. Various types of acupuncture needle manipulation techniques, including rotation and lifting/thrusting, have been passed down by practitioners over generations. However, due to the lack of objective, quantitative information concerning needle manipulation motion parameters, it is difficult for novices to acquire these sophisticated hand movements. Davis *et al*. developed a motion and force sensor to quantify different needling motion and force patterns for two different acupuncture techniques [8]. Using the *Acupuncture Manipulation Information Analysis System*, Liu et al. imitated the traditional reinforcing and reducing acupuncture manipulation described in classical Chinese medical books [9]. This system facilitates needle manipulation learning by classifying and characterizing the physical parameters of lifting-thrusting and twirling manipulations [10,11].

When learning new motor skills, observing others enables novices to acquire sensorimotor representations of the observed action [12]. However, when untrained medical students receive expert acupuncture needle manipulation instruction, imitating such complicated hand movements can prove difficult. A novel method of imitating rhythmic movements involves mimicking simulated motion pattern visual cues; VF pertaining to intended and actual movement discrepancies can be a useful tool for students [2]. Using a motion sensor device [8], we developed the *Acupuncture Manipulation Education System* (AMES), which not only simulates traditional acupuncture needle movements (using an oscillogram) but also provides information on discrepancies between this motion template and the student’s own hand movements, using concurrent or post-trial VF [13].

We herein assessed whether untrained students could improve their motor performance during acupuncture needle manipulations using AMES, with concurrent or post-trial VF (session 1: simple lifting/thrusting needle manipulations; session 2: complicated lifting/thrusting needle manipulations).

## Materials and Methods

### Participants

The study participants were 21 right-handed students from the College of Korean Medicine, Kyung Hee University, recruited using poster advertisements placed on bulletin boards. All participants had less than 4 years of medical education, with no formal education in acupuncture needle manipulation. Participants received a detailed explanation of the study, and written informed consent was obtained; they were informed that no specific risks or benefits would result from their participation. All the experiments in this study were conducted in accordance with the guidelines for human subjects committee and approved by the Institutional Review Board of Korea University, Seoul, Republic of Korea.

### Apparatus and preparation

We acquired real-time motion wave data (80.3-Hz sampling rate) using a motion sensor (Acusensor2, Stromatec, Inc., VT, USA). A phantom acupoint, developed and validated in our previous studies [14,15], was manufactured using 5% agarose gel, which produces a similar needle grasp sensation and levels of biomechanical force during needle rotation [14]. In our previous study, we also demonstrated that the 5% agarose gel phantom acupoint could be useful in a phantom-based education program for student acupuncture training. After 10 training sessions, the students’ depth error and time error during rotation manipulation as well as in lifting and thrusting manipulation outcomes were significantly improved [15].

Participants were seated in a height-adjustable chair 80 cm from a computer screen, and inserted an acupuncture needle (J-type Japanese Seirin needle: 0.25 × 40 mm; Seirin, Japan) into the phantom acupoint using the motion sensor. Before the experiment commenced, participants were instructed to hold the acupuncture needle at the phantom acupoint.

### Experimental setup and procedure

To evaluate performance according to the two types of VF, participants were randomly assigned to concurrent and post-trial VF groups using the Random Allocation software package (Revolution Analytics, Mountain View, CA). Before starting the experiment, participants practiced their intended hand movements using AMES, which provided real-time information on needle movements [13].

**Fig 1:**
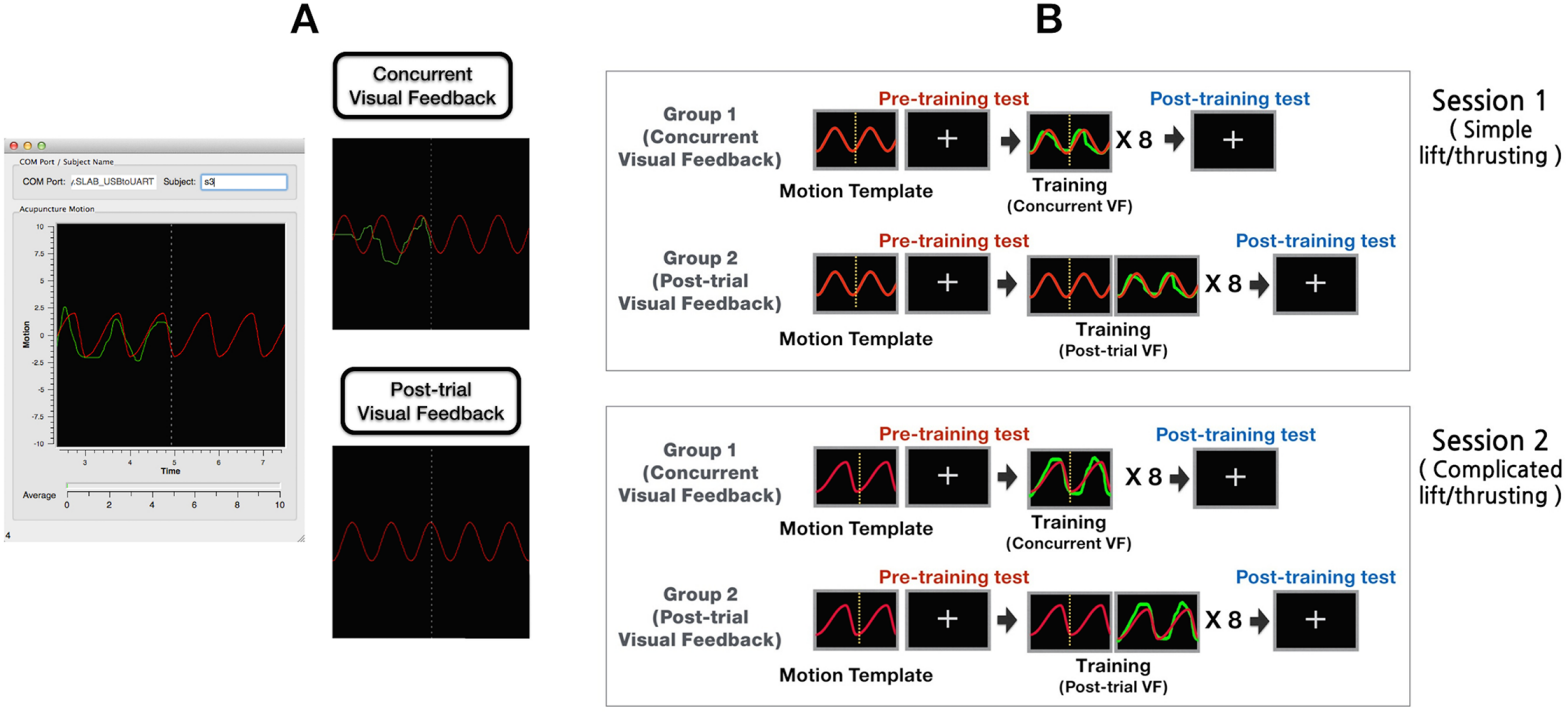
A: Example of acupuncture manipulation education system (AMES) and two different types of visual feedbacks: concurrent and post-trial visual feedback. B: Experimental setup and procedure. The experiment comprised two sessions: simple and complicated lifting/thrusting movements. Each session consisted of (1) a pre-training test, (2) training across eight trials, and (3) a post-training test. During the pre- and post-training tests, participants performed acupuncture needle manipulations without any visual information, and aimed to mimic the motion template as closely as possible (+). A simulation of their own motion (green line) overlapped the motion template (red line) in the concurrent visual feedback (VF) group; the motion information (green line) of the post-trial VF group was presented, along with the motion template (red line), immediately subsequent to each trial.

The experiment comprised two sessions (Fig 1A). In session 1, a simple lifting/thrusting movement represented the motion template (ratio of time between motions; i.e., lifting time to thrusting time = 1:1). This movement involved the isometric (strength and velocity) lifting and thrusting of a needle (movement magnitude = 4 mm, frequency = 1 Hz), such that a sine wave of 4-mm amplitude and 1-Hz frequency was produced on a moving oscillograph. In session 2, the motion template involved a complicated lifting/thrusting movement (ratio of time between motions; i.e., lifting time to thrusting time = 2:1); i.e., non-isometric (strength and velocity) lifting and thrusting of a needle (movement magnitude: 4mm, frequency: 1Hz) such that a modified sine wave of 4-mm amplitude and 1-Hz frequency was produced on a moving oscillograph. Participants rested for 15 min between the two sessions.

Each session consisted of: (1) a pre-training test, (2) training across eight separate trials, and (3) a post-training test (Fig 1B). Prior to the pre-training test, we first presented a motion template such that participants could practice hand movements. In the pre- and post-training tests, participants were instructed to perform needle manipulations, without visual information, mimicking the motion template as closely as possible. After each training test, acupuncture manipulation difficulty was rated using a numerical scale ranging between 0 (*not at all*) and 10 (*extremely*).

During the training period, both groups simulated acupuncture needle manipulation on the phantom acupoints while watching a motion template. For the concurrent VF group, a simulation of the participants’ own motion (green line) was presented, overlapping the motion template (red line). This allowed for real-time comparison with the template. For the post-trial VF group, their own motion information (green line) was presented with the motion template (red line) immediately subsequent to each trial, such that they could not detect motion errors in real-time.

During each training trial, the motion template was visualized on the AMES screen as a serial animation of a moving oscillograph; a yellow-dotted vertical line was also displayed in the center of the screen. Participants were informed that the vertical location of the point at which the moving oscillograph intersected the vertical line indicated the current position during movements. Following each training trial, participants were presented with error information (i.e., the difference between the intended and actual movements). After the experiment was complete, they were asked to evaluate the following using a 5-point Likert scale ranging between 1 (not at all) and 5 (extremely): 1) their degree of satisfaction with our education procedure and 2) its degree of necessity for formal education.

### Data processing and statistical analysis

**Fig 2:**
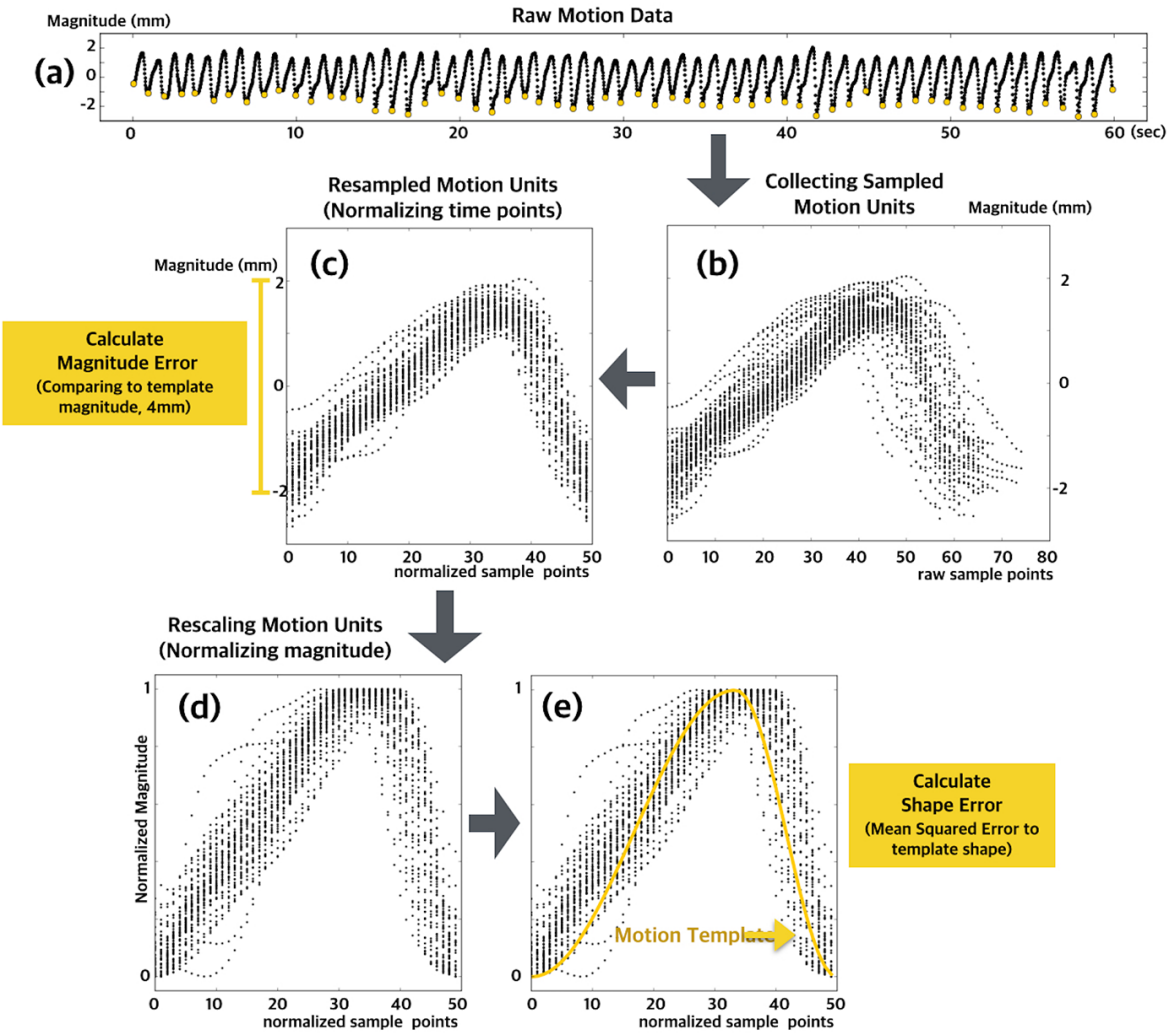
Data processing and statistical analysis. Identified sampled motion unit (a). Slicing and collection sampled motion units (b). Normalization to resample the observed data to a specific number (i.e., 50) and calculated magnitude error (c). Normalization with rescaling of the lift/thrusting amplitude to between 0 and 1 (d). Calculated shape and mean squared error (e).

For preprocessing, an infinite impulse response (IIR) Butterworth filter was applied to the raw signal as a band-pass filter, to filter out fluctuating low- (<0.2 Hz) and high-frequency noise signals (>5 Hz). Filtered raw signals were separated into repeated motions using relatively lower points as delimiters (Fig 2A). To normalize duration, we applied the resampling method to transform the observed data for each motion unit to a specific number (i.e., 50; Fig 2B). Before rescaling, the magnitudes of the stored procedures were compared to the target magnitudes (4 mm) to produce the error magnitude; absolute deviations of motion units from the target magnitude (4 mm) were averaged for each participant, and used in the group analysis (Fig 2C).

Because motion pattern is an important factor when evaluating acupuncture needle manipulation, we calculated motion errors vs. the appropriate template. To normalize amplitude, we rescaled the lifting/thrusting amplitude of each motion unit to between 0 and 1 (Fig 2D). The mean squared error of each motion unit was calculated, averaged, and used in the group analysis (Fig 2E). Data processing was conducted using the Python SciPy software package (http://www.scipy.org). To visualize representative motion patterns for each participant and group, we employed a generalized additive mixed modeling (GAMM) method using the mgcv R package (http://www.cran.r-project.org), calculating the estimated regression curve of each participant, group, and session.

We primarily compared motion patterns and error magnitudes for the pre- and post-training tests of each session for both groups. Data are presented using a box-plot. Data normality was assessed using the Shapiro-Wilk normality test. The Wilcoxon signed-rank test was used when the normality assumption was violated; otherwise, paired *t*-tests were used to test the null hypotheses that no change from the pre-training test occurred in each session and within each group, with respect to subjective test difficulty test ratings and shape error magnitudes. Statistical analyses were performed using the R software package (R Development Core Team, 2005, http://www.R-project.org). A value of *p* < 0.05 was considered to indicate statistical significance.

## Results

### Subjective difficulty ratings

No participants had previously received acupuncture needle manipulation training in either the concurrent (male: *n* = 5; female: *n* = 5; mean age = 21.8 years [*SD* = 0.9]) or post-trial (male: *n* = 4; female: *n* = 7; mean age = 21.5 years [*SD* = 1.6]) VF groups. All participants were students majoring in Korean medicine, and were in their first to fourth years of their undergraduate Korean Medicine courses, which is a 6-year curriculum. The average time spent learning basic acupuncture theory was 2.30 ± 0.27 years in the concurrent VF group and 2.40 ± 0.34 years in the post-trial VF group. None of the participants had prior experience with any practical training in acupuncture manipulation techniques.

Following AMES training, subjective difficulty ratings for the whole session decreased in both groups. In the concurrent VF group, subjective difficulty ratings were significantly reduced in sessions 1 (paired *t*-test: 6.7 ± 0.5 vs. 4.7 ± 0.5, *t* = 4.743, *p* < 0.001) and 2 (paired *t*-test: 8.6 ± 0.4 vs. 6.5 ± 0.8, *t* = 3.280, *p* < 0.01). For the VF group, post-trial subjective difficulty ratings were significantly reduced in sessions 1 (paired *t*-test: 8.1 ± 0.4 vs. 4.2 ± 0.7, *t* = 6.871, *p* < 0.001) and 2 (paired *t*-test, 8.5 ± 0.6 vs. 5.7 ± 0.8, *t* = 5.218, *p* < 0.001).

### Motion pattern analysis during acupuncture needle manipulation

**Fig 3:**
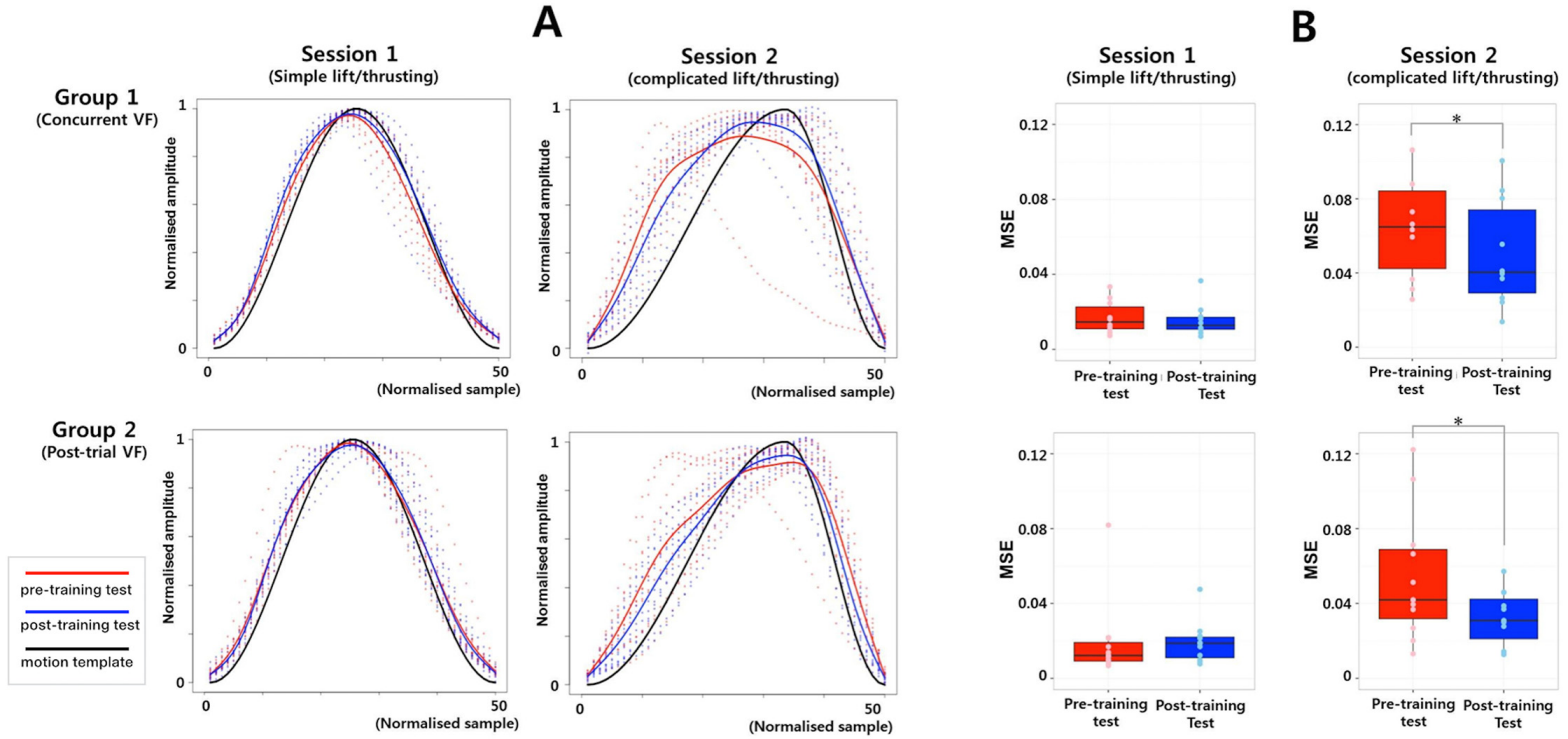
A: Estimated regression curve for the motion patterns of each participant during acupuncture needle manipulation (red dot: pre-training test, blue dot: post-training test). Mean response curve of the motion patterns of each group (red line: pre-training test, blue line: post-training test, black line: a motion template for the lifting/thrusting movement). Both the concurrent and post-trial VF groups exhibited a greater number of deviations from the motion template during the pre-training test (red line) and fewer deviations during the post-training test (blue line) in session 2. B: Both the concurrent and post-trial VF groups exhibited greater motion pattern improvements during the complicated, but not the simple, lifting/thrusting session.

Based on GAMM, we estimated true regression curves for the motion patterns of each participant during needle manipulation (red dot: pre-training test, blue dot: post-training test). We calculated the mean response curve of motion patterns for each group (red line: pre-training test, blue line: post-training test, black line: a motion template for the lifting/thrusting movement). Both groups exhibited greater deviations from the motion template during the pre-training (red line) vs. post-training (blue line) test of session 2 (Fig 3A).

During session 1, there were no significant reductions in shape errors following AMES training in either the concurrent (Wilcoxon signed-rank test, *p* = 0.492) or post-trial (Wilcoxon signed-rank test, *p* = 0.320) VF groups. However, there were significant reductions in shape errors following AMES training during session 2, in both the concurrent (Wilcoxon signed-rank test, *p* < 0.05) and post-trial (paired *t*-test, *t* = 2.506, *p* < 0.05) VF groups (Fig 3B).

### Magnitude error analysis during acupuncture manipulation

**Fig 4:**
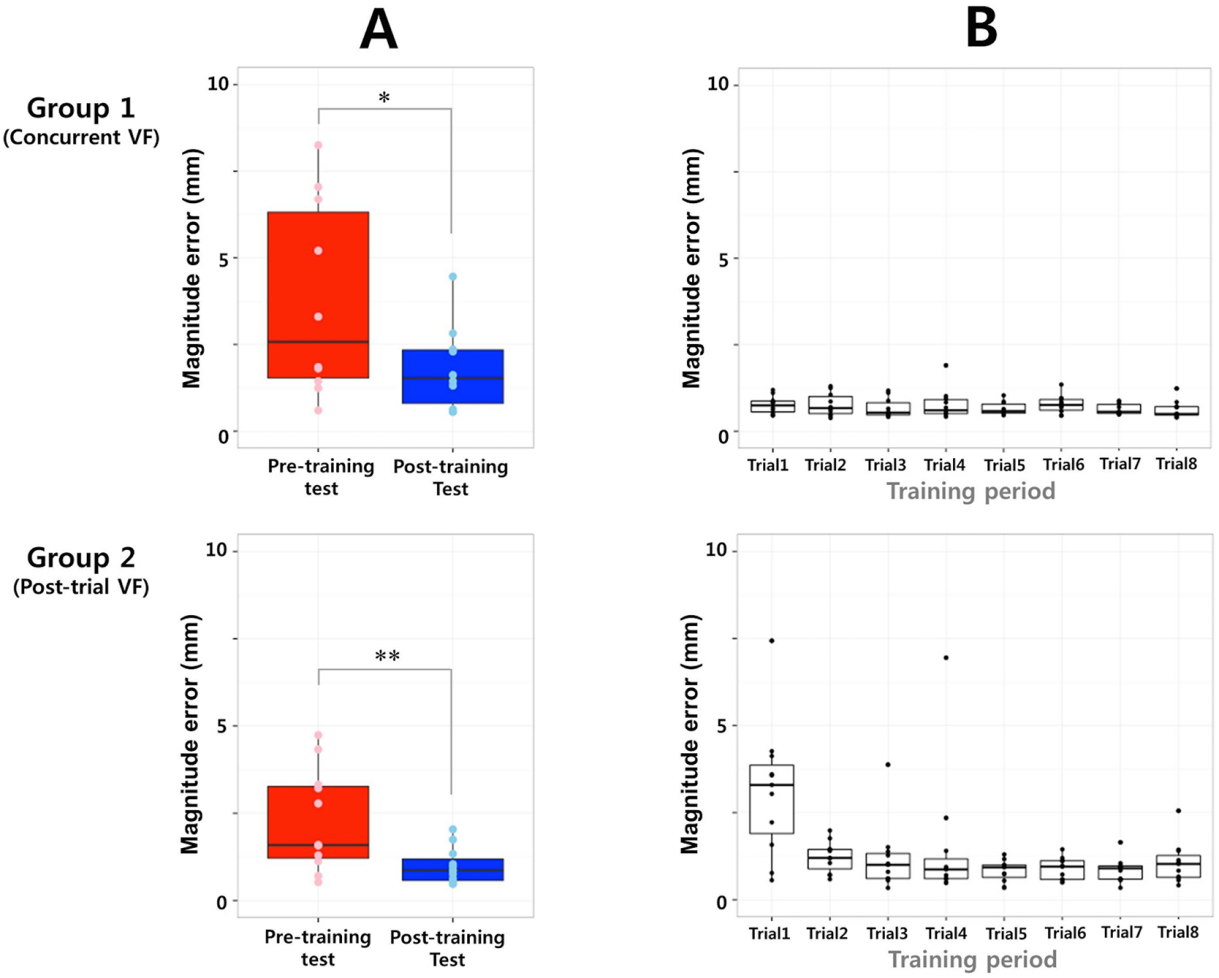
A: In the magnitude error analysis, both the concurrent and post-trial VF groups exhibited greater error reductions during the simple lifting/thrusting session. B: During the training period, the concurrent VF group exhibited reduced error magnitudes across all trials, while the post-trial VF group exhibited greater error magnitudes during the initial trials, which gradually decreased in later trials.

The magnitude error represents the absolute deviation of the needle lifting/thrusting movement from the motion template. During session 1, there were significant reductions in error magnitudes following AMES training in both the concurrent (paired *t*-test, *t* = 2.694, *p* < 0.05) and post-trial (paired *t*-test, *t* = 3.628, *p* < 0.01) VF groups (Fig 4A). During session 2, there was also a significant reduction in error magnitudes following AMES training in the concurrent VF group (Wilcoxon signed-rank test, *p* < 0.01), but not in the post-trial VF group (Wilcoxon signed-rank test, *p* = 0.320).

During the training period, the concurrent VF group exhibited reduced error magnitudes across all training trials, while the post-trial VF group exhibited greater error magnitudes during the initial training trials, which was gradually reduced in the late training trials (Fig 4B).

## Discussion

Using AMES, we provided information on participants’ hand movements, with concurrent or post-trial VF, during imitation of a motion template of traditional acupuncture needle manipulations. Following training, both the concurrent and post-trial VF groups were characterized by reduced subjective difficulty ratings across the whole session, with hand movement motion patterns more similar to the template motion pattern during the complicated lifting/thrusting session and significantly reduced error magnitudes during the simple lifting/thrusting session.

Motion pattern analysis revealed improved motion patterns during the complicated lifting/thrusting session for both groups. Because we intended to enhance hand movement performance without using simulated visual cues, we compared the needle manipulation motion patterns of the pre- and post-training tests of each group. Using GAMM, we estimated the motion pattern regression curves of each participant during the lifting/thrusting manipulation, and calculated shape error (i.e., the discrepancy between the intended and actual movements). Motion pattern analysis revealed that the estimated motion pattern regression curves were closer to the motion template in the post- vs. pre-training test of session 2 in both groups (Fig 3A). Moreover, significant shape error reductions were observed in both groups, particularly in the complicated lifting/thrusting session, following training (Fig 3B). These results suggest that acupuncture needle manipulation can be improved without visual aids following AMES training. However, shape errors were not reduced in either the concurrent or post-trial VF group during the simple lifting/thrusting session following training. Because performance of needle lifting/thrusting proved straightforward even to untrained students, there may have been a ceiling effect with respect to reductions in shape errors during the simple lifting/thrusting session.

Both the concurrent and post-trial VF groups were characterized by reduced error magnitudes during the simple lifting/thrusting session (Fig 4A). Although both groups exhibited significantly fewer errors following training, there were group differences in learning curve error magnitudes across the whole training period (Fig 4B). Because concurrent VF can provide visual information for eye-hand synchrony during practice trials, fewer magnitude errors were detected across the whole training period in the concurrent VF group. In contrast, the post-trial VF group exhibited greater error magnitudes in the initial practice trials, which decreased gradually during the later practice trials. With no real-time VF, participants in the post-trial VF group might have acquired a cognitive strategy based on the post-trial error information provided during the during practice trials [16]. Therefore, it is possible that both the concurrent and post-trial VF groups could have enhanced their motor performance using two different routes (i.e., implicit [automatic] and explicit [cognitive] processes).

Visuomotor adaptation suggests that humans largely depend on visual information to guide hand movements toward targets [2]; our model takes the form of a visuomotor map transforming visual information into motor commands [17]. Despite the >15-min rest period between the two sessions, participants in the concurrent VF group could have habituated to the visual tasks of the previous simple lift/thrusting session (practice trials and post-training test) during the pre-training complicated lift/thrusting session. There was no significant difference in the shape errors of the concurrent and post-trial VF groups during the pre-training test of session 1 (i.e., the simple lifting/thrusting session), but a greater number of shape errors were committed by the concurrent vs. post-trial VF group during pre-training for session 2 (i.e., for the complicated lifting/thrusting session). These results are consistent with previous studies in which groups receiving concurrent VF during task execution exhibited classic aftereffects, whereas groups receiving only post-trial VF did not [2]. It may be that implicit adaptation to simple lifting/thrusting manipulations during session 1 represents automatic adaptations in the concurrent VF group, whereas cognitive processes could be involved in explicit strategies for more complicated tasks in the post-trial VF group. However, to further investigate the possible effects of visuomotor adaptation, more research is required.

**Fig 5:**
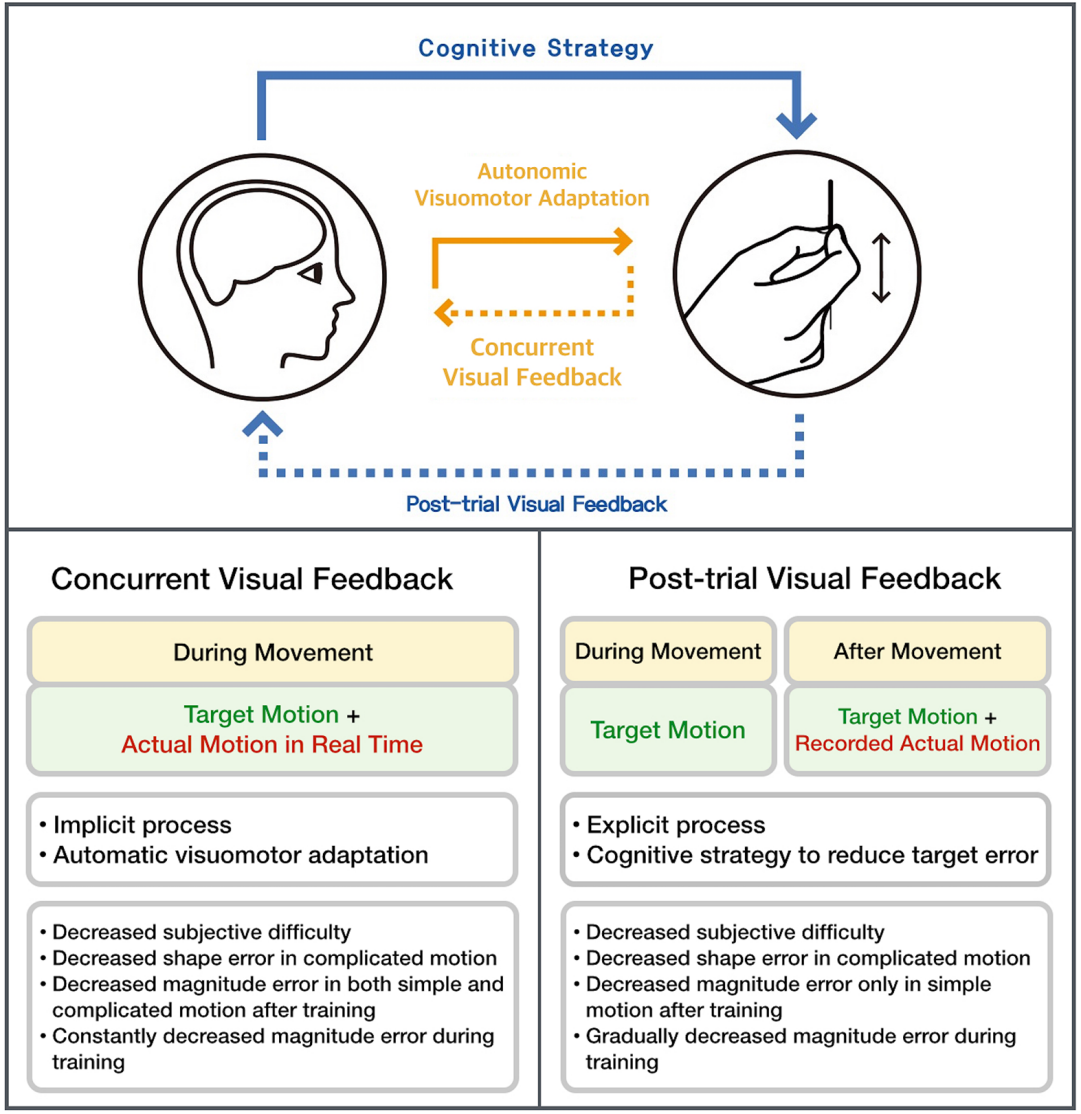
The characteristics of both conditions (concurrent and post-trial VF) are summarized. The modality, latent process, and results of each condition are included to facilitate understanding of each visual feedback condition.

Using AMES, we attempted to determine an efficient procedure for learning a very sophisticated hand movement involved in acupuncture manipulation. First, we measured acupuncture vertical movements as displayed on an oscillogram, which allows for a more precise assessment of subtle acupuncture movements than is possible with direct observation of the actual movement. Second, we tested two conditions: (1) concurrent visual feedback and (2) post-trial visual feedback. The target movements of sensorimotor learning under both conditions were provided in the form of an oscillogram. Following training, the results from both the concurrent and post-trial VF groups showed reduced subjective difficulty ratings across the whole session, with hand movement motion patterns more similar to the template motion pattern during the complicated lifting/thrusting session and significantly reduced magnitude of error during the simple lifting/thrusting session. However, only the concurrent VF group showed significantly reduced error magnitude during the complicated lifting/thrusting session (Fig 5). Under the concurrent visual feedback condition, superposing actual motion on the target movement provided a real-time reference for the discrepancy between the target motion and the actual motion. Such real-time referencing facilitates automatic recalibration of hand movements. Conversely, under the post-trial visual feedback condition, information regarding the discrepancy between the target motion and the actual motion was provided only after the actual movement. Post-trial visual feedback requires far more cognitive resources to recalibrate and to modify the actual motion to make it similar to the target motion compared with concurrent visual feedback [2].

This study had several limitations. First, we only assessed two lifting/thrusting needle manipulation skill levels. In the structural learning paradigm, the structure represents the relevant inputs and outputs of the system and the functional equations that relate to them, thereby explaining the learning-to-learn (meta-learning) phenomenon [1]. Learning the structure of a task or game, such as tennis, can facilitate motor performance in games with closely related structures (e.g., other racquet sports). Similarly, students trained in certain types of lifting/thrusting manipulations should be able to easily acquire novel needle manipulation techniques. Second, it is not clear if motor performance during acupuncture needle manipulation can be improved purely through movement repetition, including when no visual information is available. Because we aimed to investigate motor learning using two types of VF following AMES training, we did not include a non-VF group; a non-VF group would be required to differentiate error-based learning with visual cues from use-dependent learning. Third, participants received VF on a phantom, not a human, acupoint; even though this phantom acupoint is well-validated with respect to biomechanical force and needle grasp [14], it is not certain that phantom acupoint needle manipulations are fully applicable to human acupoints. However, we suggest that visuomotor training with AMES using the phantom acupoint would prove beneficial for novices learning sophisticated acupuncture hand movements prior to clinical practice. Finally, participants were presented with a motion template, using sine-wave visual cues, for both simple and complicated lift/thrusting needle manipulations. Although such visual cues are applicable to different types of lift/thrusting movements, learning from them may not be intuitive. Our data further suggest that an advanced education system should be conceived. For example, additional devices could facilitate sensorimotor learning of more complex hand movements based on the recorded movements of acupuncture manipulation performed by experienced clinicians. A novel system that simulates actual hand movements using virtual reality-based VF would be one possible method for use in the future. Despite several limitations, participants of both groups reported great satisfaction with our education system (ratings of 4.00 ± 0.22 in the concurrent VF group and 4.18 ± 0.28 in the post-trial VF group on a 5-point Likert scale), and indicated that this system should be included in the formal education program (4.60 ± 0.17 in the concurrent VF group and 4.27 ± 0.31 in the post-trial VF group). Thus, we suggest that visuomotor training with AMES using the phantom acupoint would prove beneficial for novices learning sophisticated acupuncture hand movements prior to clinical practice.

In conclusion, sensorimotor learning with VF can improve the sophisticated hand movements required for acupuncture needle manipulation. Use of two types of VF can benefit untrained students, through either automatic or cognitive processes.
